# Reduced neutrophil granular proteins and post-treatment modulation in tuberculous lymphadenitis

**DOI:** 10.1371/journal.pone.0253534

**Published:** 2021-06-21

**Authors:** Gokul Raj Kathamuthu, Kadar Moideen, Rathinam Sridhar, Dhanaraj Baskaran, Subash Babu

**Affiliations:** 1 National Institutes of Health-NIRT-International Center for Excellence in Research, Chennai, India; 2 National Institute for Research in Tuberculosis (NIRT), Chennai, India; 3 Government Stanley Medical Hospital, Chennai, India; 4 Laboratory of Parasitic Diseases, National Institute of Allergy and Infectious Diseases, National Institutes of Health, Bethesda, Maryland, United States of America; The University of Georgia, UNITED STATES

## Abstract

**Background:**

Neutrophils are important for host innate immune defense and mediate inflammatory responses. Pulmonary tuberculosis (PTB) is associated with increased neutrophil granular protein (NGP) levels in the circulation. However, the systemic levels of neutrophil granular proteins were not examined in tuberculous lymphadenitis (TBL) disease.

**Methods:**

We measured the systemic levels of NGP (myeloperoxidase [MPO], elastase and proteinase 3 [PRTN3]) in TBL and compared them to latent tuberculosis (LTB) and healthy control (HC) individuals. We also measured the pre-treatment (Pre-T) and post-treatment (Post-T) systemic levels of neutrophil granular proteins in TBL individuals upon anti-tuberculosis treatment (ATT) completion. In addition, we studied the correlation and discriminatory ability of NGPs using receiver operating characteristic (ROC) analysis.

**Results:**

Our data suggests that systemic levels of NGPs (MPO, PRTN3, elastase) were significantly reduced in TBL individuals compared to LTB and HC individuals. Similarly, after ATT, the plasma levels of MPO and elastase but not PRTN3 were significantly elevated compared to pre-treatment levels. NGPs (except PRTN3) were positively correlated with absolute neutrophil count of TBL, LTB and HC individuals. Further, NGPs were able to significantly discriminate TBL from LTB and HC individuals.

**Conclusion:**

Hence, we conclude reduced neutrophil granular protein levels might be associated with disease pathogenesis in TBL.

## Introduction

Tuberculosis (TB) is a leading cause of infection with 1.2 million deaths and 10 million active cases were reported globally in the year 2019 [1]. *Mycobacterium tuberculosis* (Mtb) primarily affects the lung parenchyma (active or pulmonary TB), or involves extrapulmonary dissemination. Extra pulmonary TB (EPTB) cases represent 16% among the 7.1 million incident cases reported by World Health Organization (WHO) in the year 2019 [1]. Among them, tuberculous lymphadenitis (TBL) is the most common form with a high female to male ratio was observed [2–4]. In India, EPTB comprises 20% of all types of TB infection; whereas, TBL disease is observed in nearly 35% of EPTB cases with cervical lymph nodes (60 to 90% of cases) being the most commonly affected [5]. Both innate and adaptive immunity, especially CD4^+^ T cells and type 1 cytokines are important for host protection [6]. However, recent investigation disclosed the potential role of neutrophils in host immune response during TB infection [7, 8].

Activation of granulocytes is a hallmark of TB disease and neutrophils were assumed to play a significant role. Since, neutrophils are the most abundant polymorphonuclear (PMN) leukocytes among white blood cells and their deficit (either inherited or acquired) leads to severe infections. In addition, they also play a vital function in eliciting immunity against bacterial pathogens [9, 10]. They are the first line of immune defense which are regulated by diverse pro and anti-inflammatory cytokines, chemokines, alarmins (such as S100A8/A9 proteins) and intrinsically expressed microRNAs (such as microRNA-223) after being recruited by tissue-resident cells [11–15]. Neutrophil granules exhibit antimicrobial activity either by one of these mechanisms such as degranulation, oxidative killing of phagocytosis from infected macrophages, reactive oxygen species and neutrophil extracellular traps (NETs) and thereby eradicates an invading pathogen [16, 17]. It is also important for magnifying inflammatory responses [18]. Some of the elements which are released by neutrophils after respiratory burst are elastase, collagenase and myeloperoxidase (MPO) and they often comprehensively harm both bacterial and host cells extracellularly [19, 20].

It was also shown in zebrafish model, polymorphonuclear leukocytes has the potential ability to kill *Mycobacterium marinum* after being infected [21]. In humans, neutrophil numbers were positively correlated with active TB disease [22, 23] and able to contributes to lung pathology in animal models [24, 25]. Similarly, both MPO and eosinophil peroxidase present in the immune cells might potentially support the host isoniazid (INH) activation. This INH treatment was greatly effective in controlling the development of active TB disease by restricting LTB infection [26, 27]. Henceforth, neutrophils potentially compose an effector cell population which can undertake both antimycobacterial and immunopathological functions during active Mtb disease [28]. However, the function of neutrophil granular proteins (NGP) in TBL disease is not well-known. In addition, whether these NGPs have the ability to serve as biomarkers for TBL disease upon differentiating LTB and HCs has not been studied. Hence, we have examined the circulating levels of NGP (MPO, PRTN3 and elastase) in TBL individuals and compared them with LTB and HC individuals. Our results show that NGPs were significantly diminished in TBL individuals and their levels were reversed significantly after the completion of anti-tuberculosis treatment (ATT).

## Materials and methods

### Ethics

The present study was approved by Internal Ethics Committees (IEC) of National Institute of Research in Tuberculosis (NIRTIEC2010007) and informed written consent form was obtained from all the study individuals. The origin of the study data are given in S1 Table.

### Study subjects

Totally, 88 blood samples were collected, 44 with tuberculous lymphadenitis (TBL) and 44 with latent tuberculosis (LTB) disease. The sample size were calculated based on our previous publications [29, 30] and all the study samples were collected from Chennai, Tamil Nadu, India. TBL individuals were diagnosed as positive either on the basis of excision biopsy (i.e affected lymph nodes) or Xpert or culture positive for *Mycobacterium tuberculosis* (Mtb) in the infected lymph node tissues. During enrolment, TBL individuals did not have any previous TB complication or administered with any anti-TB treatment (ATT). TBL individuals were treated with standard ATT for 6 months (Isoniazid, Rifampicin, Ethambutol, Pyrazinamide were given for 2 months followed by Isoniazid and Rifampicin for 4 months) and upon treatment completion, blood samples were collected once again. LTB group were diagnosed based on the positivity for both tuberculin skin test (TST) and QuantiFERON TB-Gold in tube enzyme-linked immunosorbent assay (ELISA). LTB individuals were negative for sputum smear and devoid of any pulmonary symptoms and have normal chest x-ray. A positive TST result was determined as an induration of at least 12 mm in diameter to reduce false positivity due to exposure to environmental mycobacteria. HCs were defined by the negative results on both Mantoux skin test (induration diameter <5 mm upon given with 2 tuberculin units of purified protein derivative [Staten’s Serum Institute]) and QuantiFERON-TB Gold in Tube (Qiagen) assay. All individuals had been vaccinated with Calmette-Guerin; they were negative for human immunodeficiency virus infection and pulmonary tuberculosis and not under any steroidal medications.

### Plasma collection and measurement of hematological parameters

The peripheral blood (10 ml) samples were collected and plasma was separated after centrifugation at 1,460 Relative Centrifugal Force (RCF) or G-Force for 10 min at 4°C. The plasma were aliquoted and stored at -80°C until further use. The hematological parameters between the study population were measured using an AcT 5 Diff hematology analyzer.

### Measurement of neutrophil granular proteins

The circulating levels of myeloperoxidase (MPO), proteinase3 (PRTN3) (duo set kits from R&D Systems, Minneapolis, MN, USA) and elastase (Hycult biotech) were measured using ELISA. The lowest detection limit are MPO-62.5pg/ml, PRTN3-15.625pg/ml and elastase 0.4ng/ml.

### Statistical analysis

Statistical analysis were performed using Graph-Pad PRISM (Version 8, GraphPad Software, Inc., San Diego, CA, USA). Statistical differences were evaluated using Kruskal–Wallis test as well as Wilcoxon matched-pair test and geometric means (GM) were used for the measurements of central tendency. Correlations were examined using Spearman rank correlation test. Receiver operator characteristic (ROC) analysis were carried out to test the power of each NGPs to distinguish TBL from LTB individuals. They were used to measure the sensitivity (true positives with infection) and specificity (true negatives without infection) of the TBL compared to LTB and HC individuals.

## Results

### Demographics

The detailed demographics of the study population are shown in Table 1.

**Table 1:** Demographics of the study population.

Study Demographics	TBL BL	TBL PT	LTB	HC
**No. of subjects recruited**	44	44	44	44
**Gender (Male / Female)**	21/23	21/23	22/22	23/21
**Median Age (Range)**	27 (18–51)	27 (18–51)	36 (22–65)	36.7 (19–63)
**Median Height (cm)**	160 (140–168)	160 (140–168)	163 (146–175)	161 (144–178)
**Median Weight (kg)**	45 (34–68.6)	45 (34–68.6)	59 (37–80)	62 (40–84)
**QuantiFERON-TB Gold**	Not done	Not done	Positive	Negative
**Tuberculin skin test (mm)**	Not done	Not done	<12	>12
**Absolute Neutrophil Numbers**	4002.8 (696–6879.6)	3647.58 (1227.6–6879.6)	4302.9 (2054.4–9779)	5028. 9 (2562–9439.3)

### TBL is associated with reduced neutrophil granular proteins

**Fig 1:**
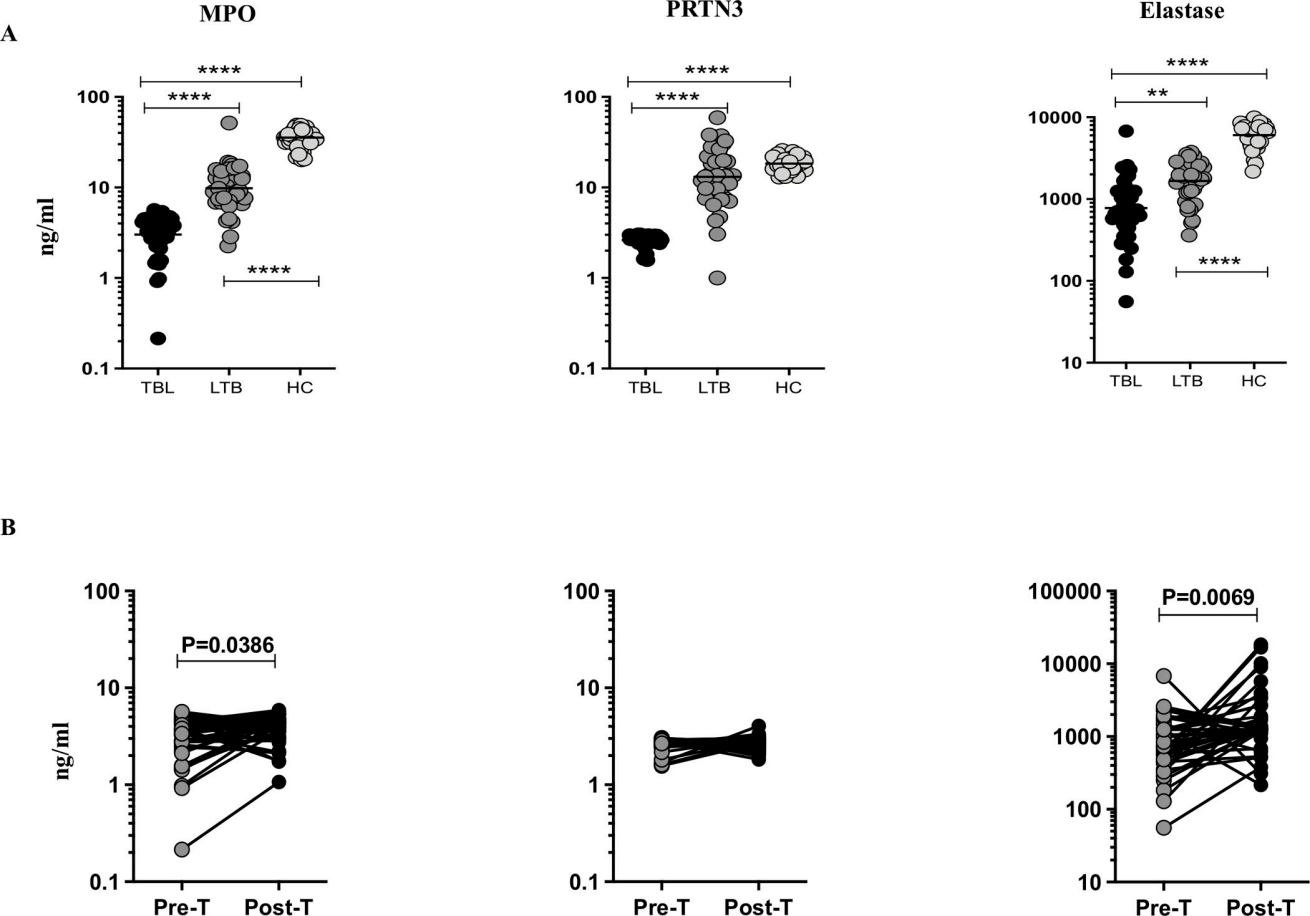
TBL is associated with diminished systemic levels of neutrophil granular proteins and reversal after the completion of ATT. (A) The circulating levels of myeloperoxidase (MPO), proteinase 3 (PRTN 3) and elastase from TBL (n = 44), LTB (n = 44) and HC (n = 44) individuals were analyzed by ELISA. Data are shown as scatter plots, with the bars representing the geometric means. P values were calculated using the Kruskal–Wallis test test. (B) The circulating levels of NGPs (MPO, PRTN3, elastase) from TBL individuals were measured at baseline (pre-treatment [pre-T]) and after the completion of 6 months of ATT (post-treatment [post-T]) by ELISA. P values were calculated using the Wilcoxon matched pair test.

To examine the systemic levels of neutrophil granular proteins in TBL, we measured the circulating levels of MPO, PRTN3 and elastase in TBL, LTB and HC individuals (Fig 1). As shown in Fig 1A, the circulating levels of MPO was significantly lower in TBL compared to LTB (GM of TBL is 3.022 ng/ml versus 9.819 ng/ml in LTB) and HC (GM of TBL is 3.022 ng/ml versus 35.45 ng/ml in HC) individuals. Similarly, the circulating levels of PRTN3 was also significantly diminished in TBL upon comparison with LTB (GM of TBL is 2.626 ng/ml versus 13.09 ng/ml in LTB) and HC (GM of TBL is 2.626 ng/ml versus 18.33 ng/ml in HC) individuals. Finally, the circulating levels of elastase was significantly diminished in TBL upon comparison with LTB (GM of TBL is 778.3 ng/ml versus 1666 ng/ml in LTB) and HC (GM of TBL is 778.3 ng/ml versus 6063 ng/ml in HC) individuals. Thus, we show TBL individuals show reduced circulating levels of NGPs as opposed to LTB and HC individuals.

### Post treatment modulation of neutrophil granular proteins in TBL

To examine the effect of anti-tuberculosis treatment (ATT) on neutrophil granular proteins (MPO, PRTN3, elastase), we have analyzed their pre and post-treatment systemic levels among TBL individuals (Fig 1B). We show after ATT completion, the circulating levels of MPO (GM of TBL pre-T is 3.022 ng/ml versus 3.722 ng/ml in post-T) and elastase (GM of PTB pre-T is 778.3 ng/ml versus 1536 ng/ml in post-T) were significantly increased in post-treatment TBL individuals compared to pre-treatment individuals. However, in contrast, we did not observe any significant changes in PRTN3 circulating levels (GM of TBL pre-T is 2.626 ng/ml versus 2.683 ng/ml in post-T) between pre and post-treatment TBL individuals. Hence, successful completion of ATT is associated with significantly enhanced circulating levels of NGPs (except PRTN3).

### Correlation analysis of neutrophil granular proteins

**Fig 2:**
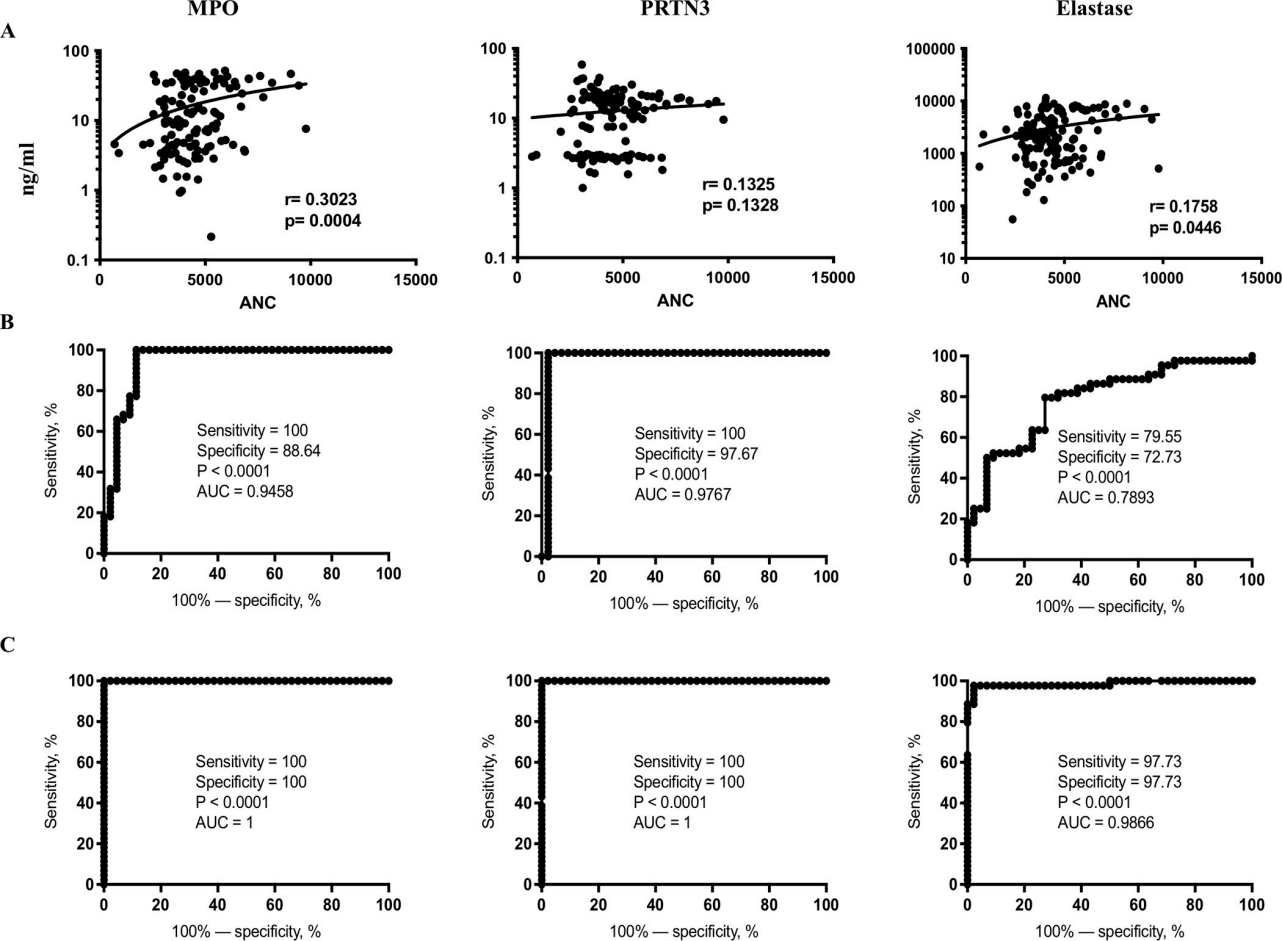
Positive significant correlation was observed among NGPs. (A) The circulating levels of NGPs (MPO, PRTN3, elastase) from all study (TBL, LTB and HC) individuals were correlated with their absolute neutrophil counts (ANC). Both r and p values were measured using Spearman rank correlation test at 95% confidence intervals. (B and C) ROC analysis (to estimate the sensitivity, specificity and area under the curve) was performed using the circulating levels of NGPs to understand the discriminatory ability to distinguish TBL from LTB and HC individuals.

Further, we also studied the association of absolute neutrophil counts with circulating levels of NGPs (MPO, PRTN3, elastase) among TBL, LTB and HC individuals (Fig 2). As shown in Fig 2A, upon correlation analysis of NGP levels with absolute neutrophil counts, MPO (r = 0.3023; P = 0.0004) and elastase (r = 0.1758; P = 0.0446)] did possess significant positive (except PRTN3) correlation between the study groups.

### ROC analysis of neutrophil granular proteins

In order to understand the discriminatory power of NGPs in discriminating TBL from LTB and HC individuals, we have executed the receiver operator characteristic (ROC) curve analysis of MPO, elastase, PRTN3 between TBL versus LTB and HC individuals. As shown in Fig 2B and 2C, proteinase 3 [sensitivity-100% and specificity-97.67%, P<0.0001, AUC = 0.9767; sensitivity-100% and specificity-100%, P<0.0001, area under the curve (AUC) = 1] showed a greatest (followed by MPO [sensitivity-100% and specificity-88.64%, P<0.0001, AUC = 0.9458; sensitivity-100% and specificity-100%, P<0.0001, AUC = 1] and elastase [sensitivity-79.55% and specificity-72.73%, P<0.0001, AUC = 0.7893; sensitivity-97.73% and specificity-97.73%, P<0.0001, AUC = 0.9866]) significant sensitivity and specificity and AUC in discriminating TBL from LTB and HC individuals.

## Discussion

Neutrophils plays an important role in initiation, regulation or suppression against both innate and adaptive immune effector functions [31–33]. They also facilitate an immune priming of Mtb via phagocytized bacteria to enter through the migratory dendritic cells and accelerates their trafficking into lymph nodes [34]. In addition, both animal and human studies indicate that neutrophil levels were correlated with disease burden and pathology, which are a common phenomenon of TB disease [35–37]. Ex-vivo analysis of neutrophil-depleted whole blood has a 3.1-fold poor ability to control Mtb infection [38, 39]. In contrast, neutrophils may also induce disease pathology through necrotic cell death rather than phagocytosis and degradation, therefore fails to kill Mtb [40, 41]. Previous data also suggest that PTB individuals are associated with enhanced systemic levels of neutrophil and eosinophil granular proteins compared to LTB individuals and significantly modulated after ATT completion indicating the role of enhanced neutrophil activity in PTB disease [42].

Myeloperoxidase (MPO) is a major protein in neutrophils and it composes 5% of the azurophilic granules [43]. It plays a significant role in respiratory burst upon neutrophil activation [43] and assists in the conversion of hydrogen peroxide (H_2_O_2_) to hypochlorous acid (HOCl) which is an important molecule for host immune defense [43, 44]. Our data show that TBL individuals exhibit significantly diminished circulating levels of MPO upon comparison with LTB and HC individuals and were modulated after the treatment completion. We therefore postulate that decreased levels of MPO could in turn favors the disease severity in TB infected individuals compared to LTB and HC groups. In addition, our data demonstrates that the plasma levels of MPO were significantly altered (elevated) upon completion of ATT in TBL individuals; suggesting Mtb bacteria might regulate the neutrophil granular proteins in TBL disease.

Similarly, our data also suggest the plasma levels of PRTN3 and elastase were significantly diminished in TBL individuals compared to LTB and HC individuals. However, in contrast, only elastase levels were significantly increased after the completion of ATT. Elastase, another crucial factor of NETs which contributes in the degradation of bacterial virulence factors and aids in the translocation of granules present in the cytoplasm to the nucleus and chromatin de condensation results in NETosis [45]. In addition, they can activate the macrophages and enhances their ability to kill intracellular pathogens and also helps in releasing increased amount of proinflammatory cytokines for protective immune response [46, 47]. Similarly, PRTN3 present in the azurophil granules of mature cell and exists in the secretory vesicles which are present near the cell surface [48, 49]. They also regulate various cytokine functions associated with metabolism as well as inflammasome complex which are responsible for elevated production and/or alteration of pro-inflammatory and decreased anti-inflammatory cytokines [50–52]. Thus, our data on PRTN3 and elastase suggests that the diminished levels of these granular proteins are a hallmark of TBL disease than LTB and HCs. Hence, we assume that PRTN3 might associate with inflammatory process; therefore, it could possibly require prolonged time to significantly modulate after treatment completion in TBL individuals.

Further, we also studied the association of absolute neutrophil counts (ANC) with NGP levels of respective TBL individuals and these proteins display significant positive association between TBL, LTB and HCs. Finally, our data show that NGPs potentially discriminate TBL from LTB and HC individuals with superior (sensitivity, specificity and AUC) distinction was observed for HCs than LTB; hence, they could act as a potential discriminatory marker. Thus, our future idea is to examine the antigen specific levels of NGPs in the affected lymph nodes of TBL individuals which possibly may disclose an essential insight into the mechanism of those markers in inducing protective or pathogenic immune responses against TBL disease.

## Supporting information

S1 Table Origin of the study data. (DOC)
