# Supplementary material for: Reduced neutrophil granular proteins and post-treatment modulation in tuberculous lymphadenitis
Source: PLoS One. 2021 Jun 21;16(6):e0253534. doi: 10.1371/journal.pone.0253534 (PMC8216526; doi:10.1371/journal.pone.0253534)
Supplement: S1 Table — (DOC) [file pone.0253534.s001.doc]

**S1 Table. Origin of the study data**

The patient samples enrolled in this present study were collected from the main study entitled “Randomized clinical trial to study efficacy and tolerability of a 4-month regimen containing ofloxacin compared to the standard 6-month regimen in treatment of patients with superficial lymph node TB”. Similarly, latent tuberculosis and healthy control samples used in this present study are recruited from the part of main study entitled “Effect of helminth Infection on antigen-specific immune responses in latent tuberculosis in South India”. The studies were approved by National Institute for Research in Tuberculosis, Internal Ethics Committee (NIRT-IEC ID-2010007; 2011013; 12I073).
